# Small-Sized Papillary Thyroid Carcinoma Mimicking a Mediastinal Lymphoma in an Adolescent With Hashimoto’s Thyroiditis: A Case Report

**DOI:** 10.7759/cureus.115150

**Published:** 2026-08-25

**Authors:** Carmen R Pozo Morales, Ronald J Alvarez Guzman, Jose M Vela, Yinno Custodio, Rolig Aliaga

**Affiliations:** 1 General Practice, Instituto de Investigaciones en Ciencias Biomédicas, Universidad Ricardo Palma, Lima, PER; 2 Oncology, Instituto de Investigaciones en Ciencias Biomédicas, Universidad Ricardo Palma, Lima, PER; 3 Oncology, Hospital de Emergencias Villa El Salvador, Lima, PER; 4 Oncohematology, Arzobispo Loayza National Hospital, Lima, PER; 5 Oncology, Arzobispo Loayza National Hospital, Lima, PER

**Keywords:** adolescent, hashimoto’s thyroiditis, lymph node metastasis, mediastinum, papillary carcinoma thyroid

## Abstract

Papillary thyroid carcinoma (PTC) is an uncommon neoplasm in pediatric and transitional age groups, characterized by a biologically more aggressive local behavior than in adults. Although its coexistence with Hashimoto's thyroiditis (HT) is widely documented, the impact of this chronic inflammation on massive lymphatic dissemination remains a subject of debate.

We present the case of a 17-year-old male who presented with cervical lymphadenopathy and computed tomography imaging suggestive of primary superior mediastinal involvement (prevascular and pretracheal). Initial fine-needle aspiration biopsy (FNAB) of the right cervical lymph node revealed atypical follicular cells with nuclear grooves, consistent with metastatic PTC. The patient underwent total thyroidectomy combined with modified bilateral radical neck dissection and central compartment neck dissection. Definitive histopathological examination confirmed a multifocal PTC (classic type and follicular variant) measuring 1.1 cm in its largest diameter, with lymphatic invasion on a background of HT (pathological staging pT1b pN1b pMx). Compartmental lymph node analysis revealed a massive metastatic burden, with 13 of 39 lymph nodes exhibiting bilateral involvement, highlighted by a 4.0 cm macrometastasis in the right middle jugular chain. Following surgery, adjuvant radioactive iodine (I-131) ablation therapy was administered after thyroid hormone withdrawal, and subsequent post-ablation follow-up laboratory testing confirmed severe endogenous hypothyroidism with a markedly elevated thyroid-stimulating hormone (TSH) level.

This clinical case highlights the striking clinicopathological discrepancy that can exist between the small size of a primary thyroid tumor and the potential for disproportionate lymphovascular dissemination in the adolescent population. When encountering bulky mediastinal and cervical masses that mimic lymphomas, PTC must be considered a critical differential diagnosis, justifying comprehensive preoperative mapping and an aggressive compartmental surgical approach.

## Introduction

Differentiated thyroid carcinoma is an uncommon neoplasm in pediatric and transitional age groups; however, it represents the most common malignant endocrine neoplasm in patients under 18 years of age, and also for approximately 8% to 10% of all pediatric head and neck cancers [1]. Within this group, papillary thyroid carcinoma (PTC) is the predominant histological variant, comprising more than 90% of reported cases [2].

Despite sharing histopathological similarities with adult presentations, PTC in children and adolescents exhibits markedly distinct biological, molecular, and clinical characteristics [3]. In the pediatric population, this neoplasm is characterized by a higher cellular proliferation rate, an elevated frequency of chromosomal rearrangements (such as RET/PTC fusions) rather than isolated point mutations like BRAF V600E, and a locally more aggressive behavior [4]. At the time of diagnosis, up to 80% of young patients present with regional lymph node metastases, and between 10% and 20% present with distant metastases, predominantly in the lungs [5]. Paradoxically, despite this marked propensity for early lymphatic dissemination, the overall long-term prognosis remains excellent, with survival rates exceeding 95% at 20 year follow-up under optimal multidisciplinary management [5,6].

The coexistence of PTC and Hashimoto's thyroiditis (HT) is well documented, though its precise prognostic impact remains debated [7]. Pathophysiologically, chronic parenchyma destruction in HT causes elevated thyroid-stimulating hormone (TSH) levels, which may act as a growth factor for atypical follicular cells [8]. Additionally, the associated inflammatory microenvironment, characterized by pro-inflammatory cytokines and reactive oxygen species, is hypothesized to facilitate extracellular matrix remodeling and tumor progression [9,10]. However, these mechanisms represent ongoing areas of investigation rather than definitive causal pathways.

The novelty of this case lies in the marked discrepancy between a small primary tumor (≤2 cm, pT1b) and extensive regional lymph node involvement extending into the superior mediastinum (pN1b, 13/39 positive nodes, including a 4.0 cm macrometastasis). Initially mimicking a primary mediastinal lymphoma or germ cell tumor, this presentation highlights the disproportionate lymphovascular aggressiveness that small PTCs can exhibit in adolescents, underscoring the necessity of comprehensive preoperative mapping and thorough surgical management regardless of primary tumor size [11,12].

## Case presentation

A 17-year-old male patient, residing in Lima, Peru, presented with a non-contributory medical history, prior exposure to ionizing radiation to the head or neck, or known family history of thyroid neoplasms or endocrinopathies. The patient's clinical presentation began with the gradual appearance of painless, firm, slow-growing, palpable cervical masses in the right lateral compartment, accompanied by non-specific symptoms.

**Figure 1:**
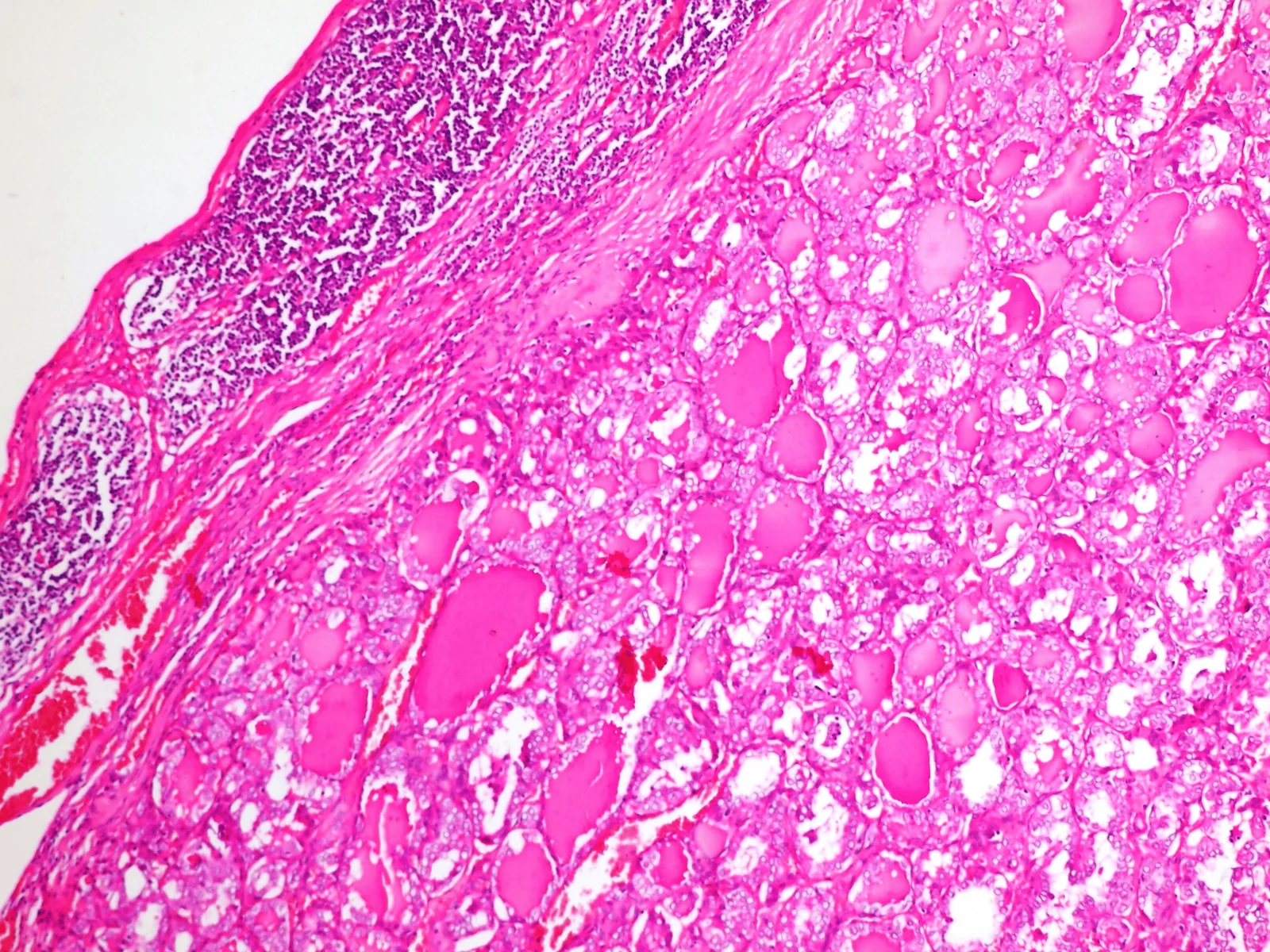
Cytology of the right cervical lymph node showing metastatic papillary thyroid carcinoma Ultrasound-guided fine-needle aspiration biopsy (FNAB) of the right cervical lymph node showing atypical, hypertrophic follicular cells with pathognomonic nuclear features of papillary thyroid carcinoma, including optically clear nuclei and longitudinal grooves.

During the initial evaluation in September 2025, an ultrasound-guided fine-needle aspiration biopsy (FNAB) of bilateral cervical lymph nodes was performed. Cytological examination of the right cervical lymph node yielded a satisfactory specimen composed of atypical, hypertrophic follicular cells exhibiting pathognomonic nuclear features: optically clear nuclei, longitudinal grooves, consistent with metastatic PTC (Figure 1). In contrast, the cytology of the left cervical lymph node was reported as negative for malignancy, showing a reactive polymorphic lymphoid population.

**Figure 2:**
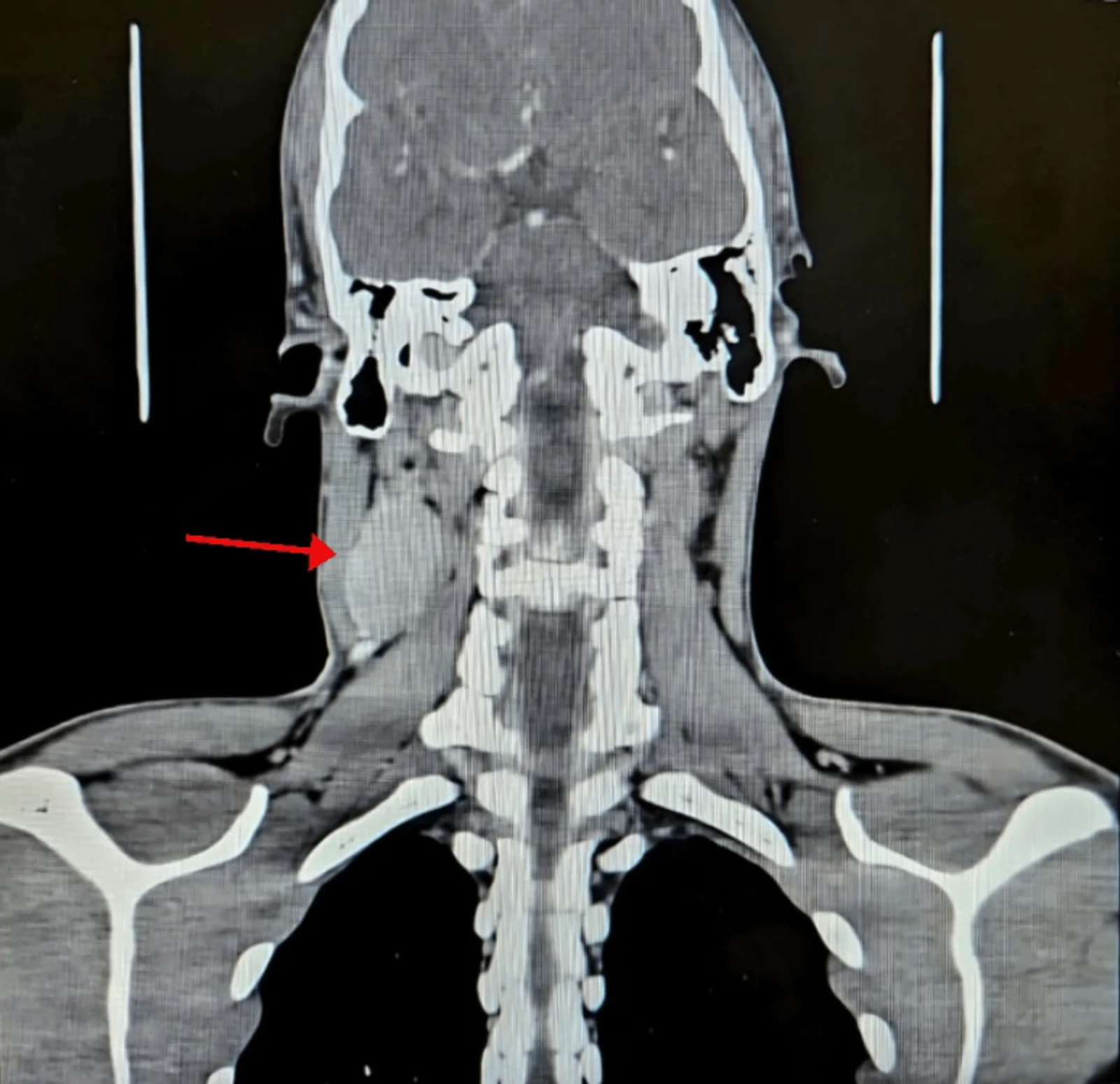
Coronal contrast-enhanced computed tomography (CT) of the neck and chest A bulky nodal mass and multiple confluent lymphadenopathies with suspicious features for secondary involvement are visualized, located in the right middle and lower jugular chain with caudal extension.

**Figure 3:**
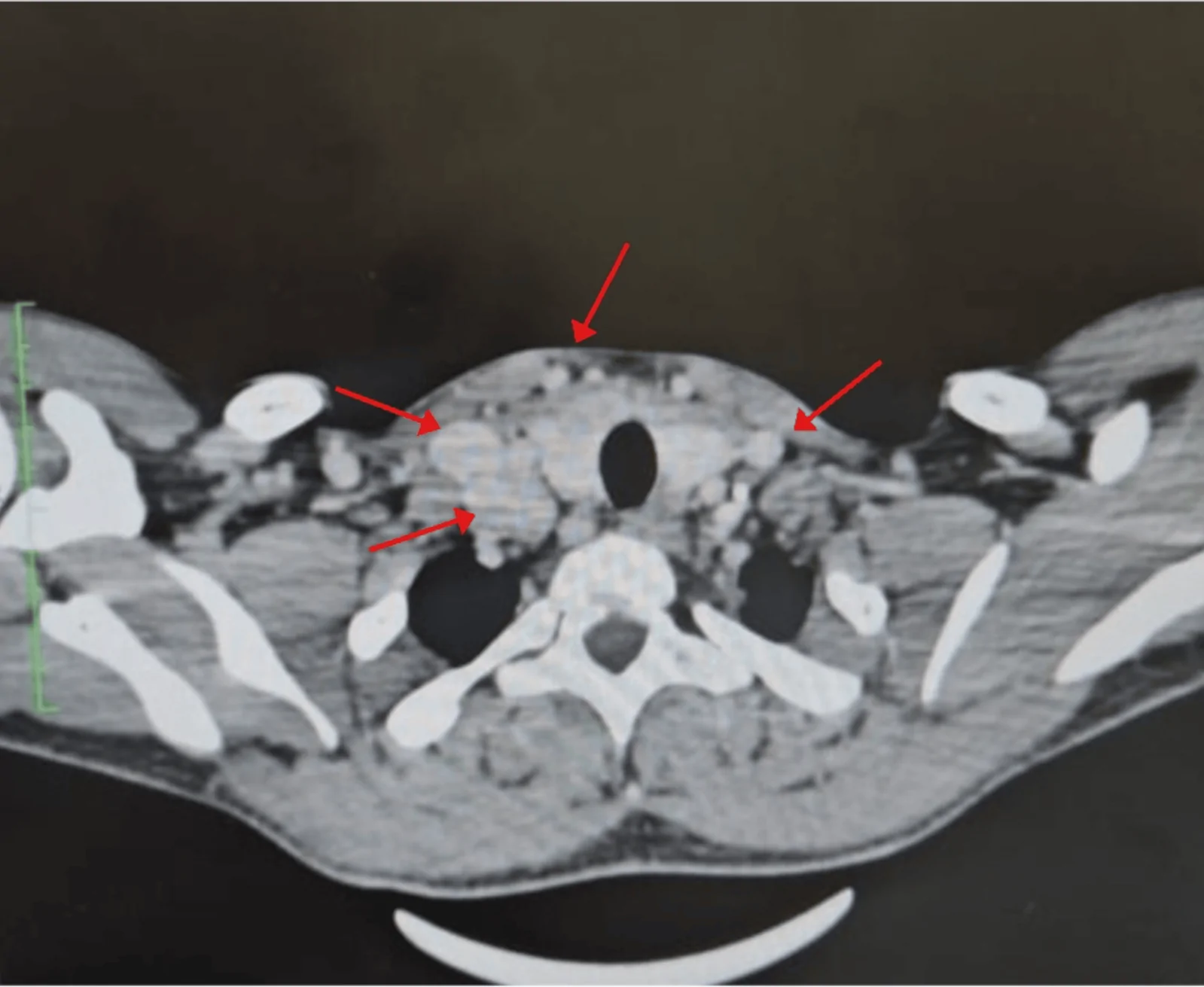
Axial contrast-enhanced cervicothoracic computed tomography (CT) at the level of the thoracic inlet Massive involvement of the central compartment is revealed, with multiple dense prevascular and pretracheal lymphadenopathies that mimic the structural radiological pattern of a primary mediastinal lymphoma or germ cell tumor.

Subsequently, to stage the extent of the disease, a contrast-enhanced chest computed tomography (CT) scan was ordered (October 2025). The imaging study revealed multiple lymphadenopathies suspicious for secondary involvement in the superior mediastinum, specifically located in the right prevascular region, measuring 20 × 17 mm, and in the pretracheal region (Figures 2-3). The lung parenchyma was properly aerated, with no nodules or consolidations, and the vascular, cardiac, and upper abdominal structures showed no abnormalities.

Given the findings of cervical and mediastinal lymphatic dissemination, the patient was scheduled for radical surgical management in December 2025. Total thyroidectomy with extensive compartmental lymphadenectomy was performed, comprising modified bilateral radical neck dissection and central compartment neck dissection.

**Figure 4:**
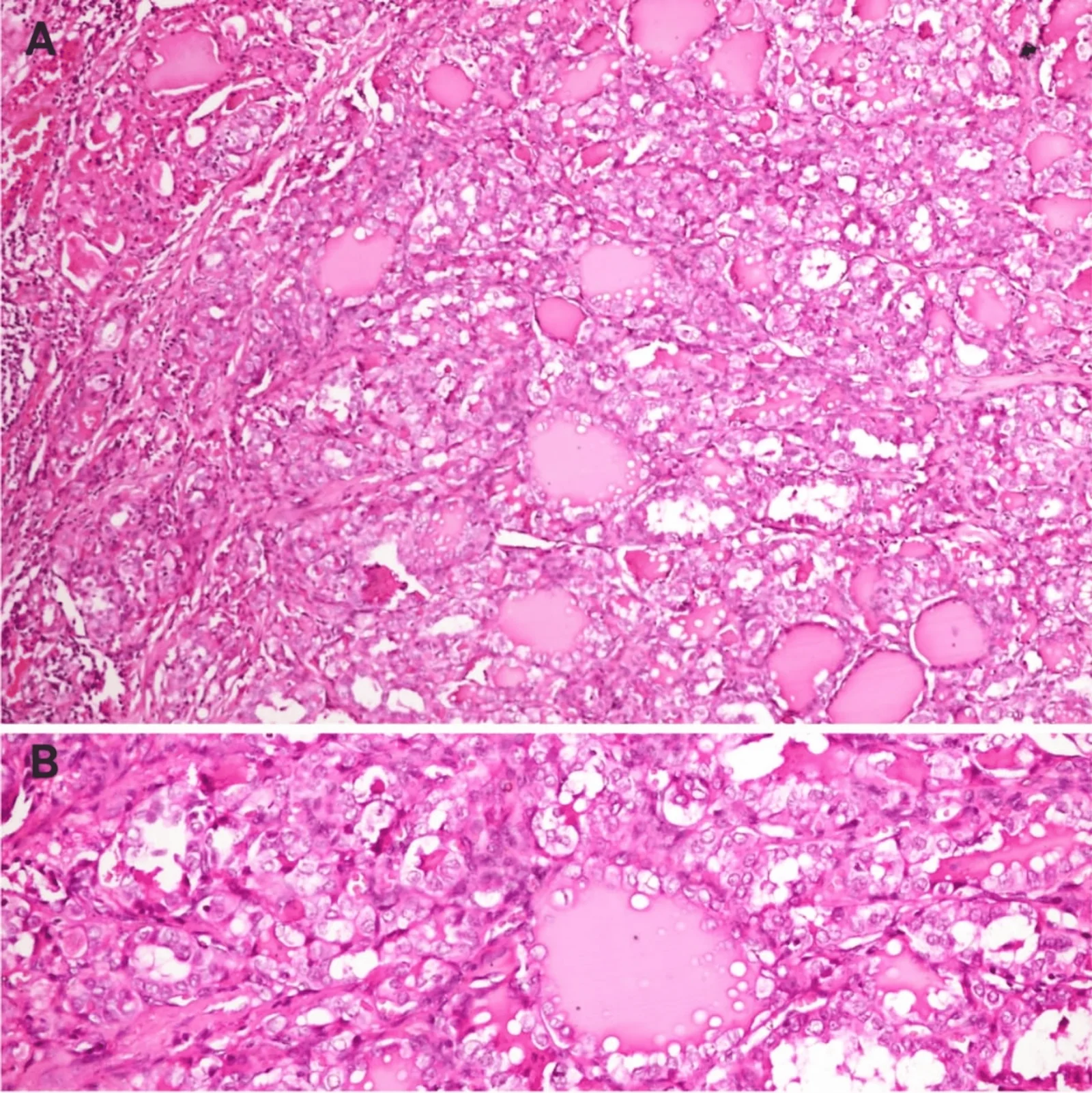
Histopathological findings of the primary papillary thyroid carcinoma (H&E stain) Histological section demonstrating a thyroid neoplasm with a mixed growth pattern featuring predominant follicular structures alongside classic papillary architectural areas. The atypical follicular cells display characteristic nuclear features of papillary thyroid carcinoma, including enlarged overlapping nuclei, optically clear chromatin ("Orphan Annie eye" appearance), nuclear grooves, and prominent intranuclear pseudoinclusions. (A) Low-power view (10×) and (B) high-power view (40×) demonstrating tumor architecture and characteristic nuclear features.

The maximum diameter of the primary tumor was 1.1 cm (located in the isthmus). Definitive histopathological analysis of the surgical specimen and subsequent institutional slide review confirmed the following findings in the thyroid gland: multifocal PTC involving the right lobe and the isthmus, exhibiting a combination of classic pattern and follicular variant (Figure 4). Lymphatic invasion and multiple psammoma body calcifications were identified. No venous vascular invasion, perineural invasion, or gross extrathyroidal extension was observed. All surgical margins were reported negative for neoplasm. The non-tumoral adjacent parenchyma demonstrated a dense chronic lymphoid infiltrate with germinal center formation, consistent with underlying HT. 

Compartmental lymph node mapping included a total of 39 lymph nodes that were isolated and examined, demonstrating a massive, bilateral metastatic burden with 13 positive lymph nodes. In the right lateral compartment, Level IIA was involved (1/5), Level IIB was negative (1/6), Level III showed complete tumor replacement and a 4.0 cm macrometastasis (4/12), Level IV was involved (0/1), and Level VB was involved (1/6). In the central and mediastinal compartment, the perithyroidal node was positive (1/1), the right paratracheal dissection showed total involvement (3/3), and the left pretracheal and paratracheal dissection showed bilateral involvement (2/5). No extranodal extension was identified. The final pathological staging was established as pT1b pN1b pMx.

In late February 2026, due to nonspecific postoperative imaging findings in the surgical beds, ultrasound-guided follow-up FNAB cytologies of both the right and left cervical beds were negative for malignancy (benign, Sydney system). Subsequently, control laboratory investigations in March 2026 documented a favorable initial biochemical profile under levothyroxine suppression, showing a basal thyroglobulin (Tg) level of 0.95 ng/mL and anti-thyroglobulin antibodies (anti-Tg) at 2.40 IU/mL (Table 1).

**Table 1:** Chronological profile of biochemical and endocrine parameters Chronological evolution of biochemical and endocrine parameters. Values marked with "-" were not requested on that date. BUN, blood urea nitrogen; PTH, parathyroid hormone; TSH, thyroid-stimulating hormone

Parameter	March 2026	May 2026	Reference Values
Ionic Calcium	-	1.10 mmol/L	1.12-1.23 mmol/L
Total Calcium	-	9.4 mg/dL	8.60-10.20 mg/dL
Serum Creatinine	0.96 mg/dL	1.05 mg/dL	Male: 0.7-1.3 mg/dL
Vitamin D Dosage (25 Hydroxy)	-	25.46 ng/mL	Insufficient: 20-<30 ng/mL
Phosphorus	-	5.0 mg/dL	Adults: 2.5-5 mg/dL
Magnesium	-	2.0 mg/dL	1.6-2.6 mg/dL
Parathyroid Hormone (PTH)	-	21.60 pg/mL	12-88 pg/mL
Total Proteins	-	7.70 g/dL	6.50-8.30 g/dL
Albumin	-	4.64 g/dL	3.50-5.20 g/dL
Globulin	-	3.06 g/dL	2.50-3.00 g/dL
Free T4	-	1.04 ng/dL	0.61-1.12 ng/dL
TSH	-	49.72 IU/mL	0.38-5.33 UI/mL
Anti-thyroglobulin	2.40 IU/mL	-	<4 IU/mL
Thyroglobulin	0.95 ng/mL	-	1.59-50.03 ng/mL
Urea	22 mg/dL	-	15-54 mg/dL
BUN	10.24 mg/dL	-	7-25 mg/dL

Following surgery, the patient was initially started on levothyroxine replacement therapy. To prepare for radioiodine treatment, levothyroxine was subsequently withdrawn for four weeks, and adjuvant radioactive iodine (I-131) ablation therapy was administered in April 2026 at a dose of 100 mCi. Subsequent follow-up laboratory testing in May 2026 confirmed a markedly elevated TSH level, documenting the severe, therapeutically induced hypothyroid state required for effective radioiodine uptake. As part of routine post-thyroidectomy surveillance, a full calcium panel was ordered; this revealed normal total calcium but a mild downward trend in ionized calcium with normal parathyroid hormone. The asymptomatic, borderline low ionized calcium was managed conservatively with low-dose oral calcium carbonate supplementation. Additionally, vitamin D insufficiency was identified and therapeutically corrected during outpatient follow-up.

The patient demonstrated excellent adherence to hormone replacement and ablative therapies, expressing a positive perspective regarding his recovery upon understanding the nature of his condition. Written informed consent was obtained from the legal guardians for publication of this case report and any accompanying medical images.

Table 2 provides a comprehensive summary of the chronological clinical milestones, diagnostic evaluations, interventions, and outcomes throughout the treatment course of patients with PTC.

**Table 2:** Clinical timeline of the case FNAB, fine-needle aspiration; I-131, radioactive iodine; PTC, papillary thyroid carcinoma

Date	Clinical Milestone/Diagnosis	Intervention/Outcome
September 2025	Onset of palpable cervical mass	Ultrasound-guided FNAB of right cervical lymph node showing atypical follicular cells consistent with metastatic papillary thyroid carcinoma.
October 2025	Radiological staging	Imaging revealed prevascular and pretracheal cystic lesions suspicious for lymphomatous involvement versus metastatic disease.
December 2025	Definitive surgical treatment	Total thyroidectomy, modified lateral neck dissection, and central compartment dissection. Histopathology confirmed 1.1 cm papillary thyroid carcinoma (pT1b pN1b; 13/39 lymph nodes positive).
February 2026	Cytological evaluation of surgical bed	Fine- needle aspiration biopsy of both right and left cervical beds negative for malignancy.
March 2026	Control biochemical assessment	Documented a favorable initial biochemical profile under levothyroxine suppression, showing a basal thyroglobulin (Tg) level of 0.95 ng/mL and anti-thyroglobulin antibodies (anti-Tg) at 2.40 IU/mL.
April 2026	Preparation for radioactive iodine ablation	Preparation for I-131 therapy. Concurrent management of mild hypocalcemia and vitamin D deficiency.

## Discussion

PTC in children and adolescents exhibits a clinical paradox widely described in contemporary literature. Despite being associated with excellent long-term overall survival rates, it displays an intrinsically more aggressive biological phenotype than in adults [1]. This aggressiveness manifests as a significantly higher propensity for extrathyroidal extension and regional lymph node involvement at initial diagnosis. While clinically evident lymph node involvement is less frequent in the adult population, its incidence rises drastically in pediatric and transitional age groups [13].

The scenario of this 17-year-old male accurately exemplifies this underlying invasive behavior. Despite presenting with a primary tumor histologically classified as small or early stage by size (1.1 cm, stage pT1b), the regional metastatic tumor burden was massive, involving 13 of 39 resected lymph nodes bilaterally (pN1b). Several genomic and molecular studies suggest that this marked propensity for disproportionate lymphovascular dissemination in young patients is linked to a high prevalence of structural chromosomal rearrangements, such as RET/PTC or NTRK oncogenic fusions, which promote a highly migratory tumor phenotype [14].

The pathophysiological relationship and prognostic impact of coexisting PTC and HT remain a focus of intense debate in oncological endocrinology [15]. Classically, multiple adult series suggest that the chronic lymphoid infiltration in HT plays a local immunoprotective role, associating with smaller tumor sizes, less advanced stages, and lower recurrence rates [16]. However, in pediatric and transitional populations, this apparent benefit is not uniform; the autoimmune inflammatory microenvironment can act detrimentally as a driver of tumor progression [5].

From a molecular perspective, HT-mediated destruction of the thyroid follicular parenchyma induces a compensatory and sustained elevation of serum TSH, which chronically stimulates follicular cells harboring pre-existing genetic alterations, accelerating their proliferation and favoring tumor multifocality [17]. Furthermore, the chronic infiltrate characteristic of autoimmunity creates a stroma rich in pro-inflammatory cytokines and growth factors that induce epithelial-mesenchymal transition. This permissive environment weakens cell-cell junctions and disproportionately facilitates the permeation and migration of neoplastic cells into perithyroidal lymphatic channels [18]. This would explain why, in this case, underlying HT coexisted with massive and aggressive lymph node dissemination.

The initial clinical presentation of this case, dominated by bulky superior mediastinal lymphadenopathies demonstrated on CT, mimicked the radiological and clinical behavior of lymphomas or germ cell tumors, representing a critical diagnostic challenge for the clinician [11]. The identification of pathognomonic PTC nuclear features via FNAB of the right cervical lymph node was decisive in promptly redirecting the diagnostic approach toward a thyroid origin [6].

For the management of these advanced scenarios (pN1b), the American Thyroid Association (ATA) guidelines for children and adolescents advocate a comprehensive, radical central and bilateral lateral compartmental surgical approach, which is essential to achieve optimal cytoreduction and reduce local persistence rates. Subsequently, given the extensive regional lymph node involvement, post-surgical radioactive iodine (I-131) was indicated as adjuvant therapy to target suspected microscopic residual disease and minimize the risk of recurrence. In pediatric and transitional populations, this indication is guided by an individualized post-operative risk-benefit assessment, incorporating the extent of initial surgical resection, post-treatment imaging, and biochemical findings [19].

## Conclusions

PTC in adolescent patients can exhibit a lymphovascular aggressiveness that is markedly disproportionate to the millimetric dimensions of the primary tumor. Careful preoperative cervical lymph node mapping should therefore be considered even in apparently low-volume primary tumors in adolescents. When encountering bulky anterosuperior mediastinal masses and concurrent cervical lymphadenopathies in young patients, PTC must be considered a critical differential diagnosis. Radiological features mimicking lymphoproliferative diseases or other primary mediastinal neoplasms necessitate a meticulous diagnostic approach utilizing FNAB cytology.

## References

[1] Francis GL, Waguespack SG, Bauer AJ (2015). Management guidelines for children with thyroid nodules and differentiated thyroid cancer. Thyroid.

[2] Gagnier JJ, Kienle G, Altman DG, Moher D, Sox H, Riley D (2014). The CARE guidelines: consensus-based clinical case report guideline development. J Clin Epidemiol.

[3] Parvathareddy SK, Siraj AK, Annaiyappanaidu P (2023). Predictive risk factors for distant metastasis in pediatric differentiated thyroid cancer from Saudi Arabia. Front Endocrinol (Lausanne).

[4] Bauer AJ (2020). Pediatric thyroid cancer: genetics, therapeutics and outcome. Endocrinol Metab Clin North Am.

[5] Cinar I, Sengul I (2024). Coexistence of Hashimoto's thyroiditis and papillary thyroid carcinoma revisited in thyroidology, an experience from an endemic region: fad or future?. Rev Assoc Med Bras (1992).

[6] Salih AM, Qaradakhy AJ, Dhahir HM (2025). Papillary thyroid microcarcinoma presenting as a large cystic lymph node: a case report. Oncol Lett.

[7] Goldfarb M, Christison-Lagay E, Rastatter J, Wasserman J (2022). Young children are not the same as adolescents when it comes to treating thyroid cancer. J Clin Endocrinol Metab.

[8] Mitsutake N, Saenko V (2021). Molecular pathogenesis of pediatric thyroid carcinoma. J Radiat Res.

[9] Yao S, Zhang H (2025). Papillary thyroid carcinoma with Hashimoto's thyroiditis: impact and correlation. Front Endocrinol (Lausanne).

[10] Sacristán-Gómez P, Serrano-Somavilla A, Castro-Espadas L (2023). Evaluation of epithelial-mesenchymal transition markers in autoimmune thyroid diseases. Int J Mol Sci.

[11] Chung YS, Suh YJ (2010). Is central lymph node dissection mandatory in 2 cm or less sized papillary thyroid cancer?. J Korean Surg Soc.

[12] Ferrari SM, Fallahi P, Galdiero MR (2019). Immune and inflammatory cells in thyroid cancer microenvironment. Int J Mol Sci.

[13] Sugino K, Nagahama M, Kitagawa W (2015). Papillary thyroid carcinoma in children and adolescents: long-term follow-up and clinical characteristics. World J Surg.

[14] Dimachkieh AL, Mahajan P (2024). Management of pediatric thyroid carcinoma. Curr Opin Otolaryngol Head Neck Surg.

[15] Chen P, Yao Y, Tan H, Li J (2024). Systemic treatments for radioiodine-refractory thyroid cancers. Front Endocrinol (Lausanne).

[16] Noureldine SI, Tufano RP (2015). Association of Hashimoto's thyroiditis and thyroid cancer. Curr Opin Oncol.

[17] Khan FA, Al-Jameil N, Khan MF, Al-Rashid M, Tabassum H (2015). Thyroid dysfunction: an autoimmune aspect. Int J Clin Exp Med.

[18] Du JJ, Wang J, Ma K, Ma P (2026). New insights into the tumor immune microenvironment and immunotherapy of thyroid cancer. Front Immunol.

[19] Pavlidis ET, Pavlidis TE (2023). Role of prophylactic central neck lymph node dissection for papillary thyroid carcinoma in the era of de-escalation. World J Clin Oncol.

